# Updating the Vibrio clades defined by multilocus sequence phylogeny: proposal of eight new clades, and the description of *Vibrio tritonius* sp. nov.

**DOI:** 10.3389/fmicb.2013.00414

**Published:** 2013-12-27

**Authors:** Tomoo Sawabe, Yoshitoshi Ogura, Yuta Matsumura, Gao Feng, AKM Rohul Amin, Sayaka Mino, Satoshi Nakagawa, Toko Sawabe, Ramesh Kumar, Yohei Fukui, Masataka Satomi, Ryoji Matsushima, Fabiano L. Thompson, Bruno Gomez-Gil, Richard Christen, Fumito Maruyama, Ken Kurokawa, Tetsuya Hayashi

**Affiliations:** ^1^Laboratory of Microbiology, Faculty of Fisheries Sciences, Hokkaido UniversityHakodate, Japan; ^2^Division of Genomics and Bioenvironmental Science, Frontier Science Research Center, University of MiyazakiMiyazaki, Japan; ^3^Department of Food and Nutrition, Hakodate Junior CollegeHakodate, Japan; ^4^National Institute for Interdisciplinary Science and Technology (CSIR)Kerala, India; ^5^National Research Institute of Fisheries Science, Fisheries Research AgencyYokohama, Japan; ^6^Department of Genetics, Center of Health Sciences, Federal University of Rio de Janeiro (UFRS)Rio de Janeiro, Brazil; ^7^A.C. Unidad Mazatlán, CIADMazatlán, México; ^8^CNRS UMR 7138, Systématique-Adaptation-EvolutionNice, France; ^9^Systématique-Adaptation-Evolution, Université de Nice-Sophia AntipolisNice, France; ^10^Graduate School of Medical and Dental Sciences, Tokyo Medical and Dental UniversityTokyo, Japan; ^11^Earth-Life Science Institute, Tokyo Institute of TechnologyTokyo, Japan

**Keywords:** vibrios, *Vibrionaceae*, multilocus sequence analysis, evolution, housekeeping protein gene, *Vibrio tritonius*

## Abstract

To date 142 species have been described in the *Vibrionaceae* family of bacteria, classified into seven genera; *Aliivibrio*, *Echinimonas*, *Enterovibrio*, *Grimontia*, *Photobacterium*, *Salinivibrio* and *Vibrio*. As vibrios are widespread in marine environments and show versatile metabolisms and ecologies, these bacteria are recognized as one of the most diverse and important marine heterotrophic bacterial groups for elucidating the correlation between genome evolution and ecological adaptation. However, on the basis of 16S rRNA gene phylogeny, we could not find any robust monophyletic lineages in any of the known genera. We needed further attempts to reconstruct their evolutionary history based on multilocus sequence analysis (MLSA) and/or genome wide taxonomy of all the recognized species groups. In our previous report in 2007, we conducted the first broad multilocus sequence analysis (MLSA) to infer the evolutionary history of vibrios using nine housekeeping genes (the 16S rRNA gene, *gapA*, *gyrB*, *ftsZ*, *mreB, pyrH*, *recA*, *rpoA*, and *topA*), and we proposed 14 distinct clades in 58 species of *Vibrionaceae*. Due to the difficulty of designing universal primers that can amplify the genes for MLSA in every *Vibrionaceae* species, some clades had yet to be defined. In this study, we present a better picture of an updated molecular phylogeny for 86 described vibrio species and 10 genome sequenced *Vibrionaceae* strains, using 8 housekeeping gene sequences. This new study places special emphasis on (1) eight newly identified clades (*Damselae*, *Mediterranei*, *Pectenicida*, *Phosphoreum*, *Profundum*, *Porteresiae*, *Rosenbergii*, and *Rumoiensis*); (2) clades amended since the 2007 proposal with recently described new species; (3) orphan clades of genomospecies F6 and F10; (4) phylogenetic positions defined in 3 genome-sequenced strains (N418, EX25, and EJY3); and (5) description of *V. tritonius* sp. nov., which is a member of the “*Porteresiae*” clade.

## Introduction

Bacterial systematics has evolved alongside the development of innovative methodologies and techniques (Wayne et al., 1987; Stackebrandt et al., 2002; Gevers et al., 2005). The first definition of bacterial species in “phylogenetic terms” was developed in 1987 using the DNA-DNA reassociation and DNA sequencing. These approaches to bacterial systematics provided us with a uniform definition of prokaryotic species (Wayne et al., 1987). In 2002, an *ad hoc* committee listed additional innovative methods that could be used for bacterial systematics, such as 16S rRNA gene sequence analysis, DNA typing methods (AFLP, RAPD, Rep-PCR, PFGE), MLSA, WGS analysis, and proteomics (Stackebrandt et al., 2002). The primary purpose of the committee's statement was to promote dialogue among systematists, population and evolutionary geneticists, ecologists and microbiologists for the benefit of bacterial systematics in general, and to create a more transparent species concept in particular (Stackebrandt et al., 2002). Among those innovative methodologies, MLSA and the WGS analysis have become the most important and successful methodologies; their strong impact on bacterial systematics is due to data reproducibility and portability (see, e.g., Maiden et al., 1998; Aanensen and Spratt, 2005; Gevers et al., 2005; Konstantinidis and Tiedje, 2005; Staley, 2006; Goris et al., 2007; Richer and Rosselló-Móra, 2009; Auch et al., 2010).

MLST, the prototype for MLSA-based methodology, was used for the first highly portable typing of *Neisseria meningitides* from invasive disease and healthy carriers, and it yielded the first understanding of the epidemiology and population structure of that infectious agent (Maiden et al., 1998). Its high levels of discriminatory power between those strains, which required half the loci typically required for a classical allozyme electrophoresis, and its superior application to evolutionary, phylogenetic, or population genetic studies, allowed researchers to develop MLST schemes for a number of bacteria taxa (Aanensen and Spratt, 2005) (also refer to the MLST website; http://www.mlst.net/). It also opened the use of MLSA for bacterial systematics (e.g., Sawabe et al., 2007; Thompson et al., 2007; Bishop et al., 2009), and it guided the reconsideration and re-evaluation of prokaryotic species concepts (Gevers et al., 2005; Staley, 2006; Preheim et al., 2011). Even now, MLSA provides a better understanding of taxonomically controversial bacterial taxa; for example, the human origins of the *Agrobacterium* (*Rhizobium*) *radiobacter* clustered as a well-separated genovar (Aujoulat et al., 2011) and the highly versatile aeromonads consisting of 3 major clades (Roger et al., 2012).

In the genome era, genome sequencing has been used to characterize new bacterial species (Haley et al., 2010; Hoffmann et al., 2012), to reclassify bacterial taxons such as *Neisseria* (Bennett et al., 2012), *Acinetobacter* (Chan et al., 2012) and *Vibrio* (Lin et al., 2010), and to challenge defined prokaryotic species (Konstantinidis and Tiedje, 2005; Thompson et al., 2009; Chan et al., 2012). Using these WGSs, *in-silico* DDH calculations can also be emulated, mainly in two ways: high-scoring segment pairs (HSPs) (Konstantinidis and Tiedje, 2005; Goris et al., 2007) and the genome-to-genome distance calculation, called the Digital DDH measurement (Auch et al., 2010). The criterion of more than 95% ANI is currently a widely used similarity value for species delineation. The WGS analysis also supercedes the limitations of MLSA, which is only capable of including genes that are successfully amplified by designed primers (Gevers et al., 2005; Thompson et al., 2005; Sawabe et al., 2007).

*Vibrionaceae* are at the forefront of bacterial taxons being tested with new innovative methodologies and techniques for bacterial systematics (Thompson et al., 2001, 2005, 2009; Sawabe et al., 2007). The number of species described in *Vibrionaceae* has increased remarkably since the establishment of genome fingerprinting techniques (Thompson et al., 2001) and MLSA schemes (Thompson et al., 2005, 2007; Sawabe et al., 2007, 2009). Now, a total of 142 species are recognized in the family *Vibrionaceae* (Association of Vibrio Biologists website; http://www.vibriobiology.net/). *Vibrionaceae* are defined as a group of strains with the following characteristics: they are Gram-negative rods with a polar flagellum enclosed in a sheath, have facultative anaerobic metabolisms, are capable of fermenting D-glucose, and grow at 20°C. The bacteria are primarily aquatic, and most species are oxidase positive, can reduce nitrate to nitrite, require Na^+^ for growth, and ferment D-fructose, maltose, and glycerol (Gomez-Gil et al., 2014). In addition, most vibrio species ferment a variety of carbohydrates without gas production, and grow on TCBS medium (Farmer III et al., 2005; Thompson et al., 2009). As we experience a rapid expansion in the number of known species in the family *Vibrionaceae*, we face a number of unique vibrio isolates that lack one or more of the above common properties. *Vibrionaceae* species are metabolically versatile, and the number of species showing gas production, nitrogen fixation, phototrophy, and non-motility is increasing (Gomez-Gil et al., 2014). Considerably more attention should be paid to the biological and genetic plasticity of vibrios to help understand the dynamics of vibrio evolution (Sawabe et al., 2007; Grimes et al., 2009; Thompson et al., 2009).

Due to the limitations of using 16S rRNA gene phylogeny (Gomez-Gil et al., 2014) to elucidate an “Integrated Vibrio Biology” that includes a biodiversity assessment, an inferred evolutionary history, the population biology, and genomics, Sawabe et al. (2007) developed an MLSA scheme for the family *Vibrionaceae* using nine gene sequences (*ftsZ*, *gapA*, *gyrB*, *mreB*, *pyrH*, *recA*, *rpoA*, *topA* and the 16S rRNA gene). The analysis involved the complete sequence sets of 9 genes from 58 vibrio taxa, and it revealed 14 monophyletic clades with a significant bootstrap support. The species within each clade shared >20% DDH, <5% G+C (mol%), >85% MLSA sequence similarity, and >89% AAI (Sawabe et al., 2007).

Recent extraordinary progress in biodiversity studies and WGS projects in vibrios has resulted a substantial leap in novel vibrio species and taxonomically unassigned strains. In fact, after the proposal of 14 robust clades in 2007, more than 60 new species have been described. The number of genome-sequenced strains has exceeded 1000. It is, therefore, obvious that the phylogenetic tree based on the multilocus gene sequences reported in 2007 is insufficient to show the most recent molecular phylogenetic structure of vibrios.

In our previous analysis of multilocus gene phylogeny, eight housekeeping protein coding genes and the 16S rRNA gene were included in the MLSA. However, the 16S rRNA gene has a rather low interspecies resolution (100, 99.5, and 95.3% of maximum, median, and minimum resolution, respectively) (see Figure 5S, in Sawabe et al., 2007). It was also difficult to include the 16S rRNA gene sequences for the calculation of the radiation time of each clade. We have been debating whether the inclusion of the 16S rRNA gene sequences is necessary to infer the evolutional history of vibrios, but there are no other proper and fast tools to check the tree topologies constructed using a rather large data set (58 species).

A variety of methods have been proposed to tackle the problem of gene tree reconciliation to reconstruct a species tree. When the taxa in all the trees are identical, the problem can be stated as a consensus tree problem (Guénoche, 2013). The comparison of gene trees and their assembly into a unique tree representing the species tree is a general problem in phylogeny. However, in this study, we faced a different problem: determining whether the inclusion or exclusion of a given gene in the analysis would substantially change the outcome. This was the method used in this analysis to investigate if the inclusion or exclusion of the 16S rRNA gene sequences in the MLSA analysis would, or would not, affect the final result.

The aims of this study were to re-evaluate how the 16S rRNA gene sequence affects the final phylogenetic tree, as based on multilocus gene sequencing analysis; to update our knowledge in vibrio biodiversity and evolution on the basis of an 8-gene MLSA; and to reconstruct a better vibrio phylogeny. The analysis provided a further opportunity to propose additional eight clades to the most up-to-date vibrio phylogeny.

## Materials and methods

### Subtree incongruence test of multilocus gene phylogeny

The usual approach to compare trees is to count how many subtrees they share; a given subtree often has a different topology according to the method used (NJ, ML, or MP) but the differences are often subtle and generally not well-supported. Accordingly, it is best to compare trees according to bipartitions (a tree is considered as a set of bipartitions, each one corresponding to an internal edge of the tree, the external ones connecting the leaves to the tree) (Guénoche, 2013). This was the method used in this analysis to investigate whether the inclusion or exclusion of the 16S rRNA gene sequences in the MLSA analysis would, or would not, affect the final result. TreeDyn (Chevenet et al., 2006) was used to compare trees to subtrees sharing the same topology. A dedicated python script (using libraries from Huerta-Cepas et al., 2010) was used to compare trees with shared sub-trees, independently of their topologies.

Our previous MLSA revealed that the 16S rRNA gene, in contrast to the other gene sequences used, has a rather low interspecies resolution (Sawabe et al., 2007). To increase the sensitivity and reduce the time taken by MLSA to update the vibrio phylogeny, we compared the subtree topologies obtained from a nine-gene data set (that included the 16S rRNA gene) and an eight-gene data set (that excluded the 16S rRNA gene). We used the gene sequence data set from the 58 species, for which we had the sequence of every gene, and which is identical to the data set used in Sawabe et al. (2007). The method selected, (1) shared subtrees only if they had the same topology, and (2) subtrees that shared the same species, independently of their topology. The results were visualized using TreeDyn (Chevenet et al., 2006), and congruent subtrees with the same topologies were indicated using the same color.

### Sequencing of housekeeping protein-coding genes

An additional eight housekeeping genes of type strains of the genera *Vibrio* and *Photobacterium* were sequenced manually according to Sawabe et al. (2007) with newly designed primers (Vgap150f:ACTCAYGGYCGTTTCAACGGYAC, Vgap957r:RCCGATTTCGTTRTCGTACCAAG, VftsZf55f:GTKGGTGGCGGCGGCGGTAA, VftsZ782r:ACACCACGWGCACCAGCAA GATCG, VftsZ-9f:ACCGATGATGGAAATGTCTGACGATGC, VmreB225f:RATGAAA GACGGCGTWATYGC, VmreB1025r:TCGCCRCCGTGCATRTCGATCA) (Table S1). All strains were maintained in ZoBell 2216E agar and stored with 20% glycerol at −80°C. Whole genome sequencing was performed in nine type strains (*V. aerogenes* LMG 19650^T^, *V. gazogenes* ATCC 29988^T^, *V. halioticoli* IAM 14596^T^, *V. neonatus* HDD3-1^T^, *V. porteresiae* MSSRF 30^T^, *V. rhizosphaerae* MSSRF 3^T^, *V. ruber* LMG 23124^T^, *V. tritonius* sp. nov. AM2^T^, and *V. superstes* G3-29^T^) using the Roche 454 FLX titanium genome sequencer alone or in combination with the Illumina MiSeq sequencer. The sequence reads were assembled using the Newbler software version 2.3 or later. Illumina reads were used only for sequence error correction. After auto-annotation by Microbial Genome Annotation Pipeline (MiGAP, http://www.migap.org/; Sugawara et al., 2009), relevant housekeeping gene sequences were retrieved and used for the MLSA. The house keeping genes necessary for updating the vibrio phylogeny were also retrieved from the latest version of the NCBI microbial genome and GenBank database (Release 197.0, 15 August 2013), and used in the analysis. All sequence data used in this study are listed in Table S1.

### Sequence analysis

MLSA was performed the same way as in Sawabe et al. (2007). The sequences were aligned using the ClustalX program (Larkin et al., 2007). The domains used to construct the phylogenetic trees shown in Figures 2, 3 were regions of the *ftsZ*, *gapA*, *gyrB*, *mreB*, *pyrH*, *recA*, *rpoA*, and *topA* genes of *Vibrionaceae*: positions 195–630, 225–861, 441–1026, 390–897, 171–543, 429–915, 87–873, and 570–990 (*V. cholerae* O1 Eltor N16961 (AE003852) numbering), respectively. The regions were within those used in the previous study (Sawabe et al., 2007). Sequence similarity and the number of nucleotide and amino acid mutation were determined using MEGA version 5 (Tamura et al., 2011).

Split Decomposition Analysis (SDA) was also performed as described in Sawabe et al. (2007) using SplitsTree version 4.12.8, with a neighbor net drawing and Jukes-Cantor correction (Bandelt and Dress, 1992; Huson and Bryant, 2005). The concatenated sequences of the eight housekeeping genes were also generated using the program and used for a phylogenetic analysis combined with NJ, MP, and ML analyses (Sawabe et al., 1998).

### Phylogenetic, genetic, phenotypic, and chemotaxonomic characterization of *Vibrio tritonius* sp. nov

Four isolates of *V. tritonius* sp. nov., JCM 16456^T^ = LMG 25401^T^ = AM2^T^, JCM 16457 = LMG 25402 = MA12, JCM 16458 = LMG 25403 = MA17, and JCM 16459 = LMG 25404 = MA35, isolated from gut of a sea hare, *Aplysia kurodai*, were used in this study. The strains were cultured on ZoBell 2216E agar (Oppenheimer and ZoBell, 1952) and stored at −80°C in 10% glycerol-supplemented broth.

A total of 1400 bp 16S rRNA gene sequences of the four strains were determined according to Sawabe et al. (1998) using four sequence primers (24F, 1100F, 920R, and 1509R). The 16S rRNA gene sequences were blasted to the latest release ver. 197 of GenBank and related sequences were retrieved. Finally, 16S rRNA gene sequences of *V. aerogenes* X74705, *V. brasiliensis* AJ316172, *V. cholerae* X76337, *V. fluvialis* X74703, *V. furnissii* X76336, *V. gazogenes* X74705, *V. hepatarius* AJ345063, *V. nereis* X74716, *V. porteresiae* EF488079, *V. rhizosphaerae* DQ847123, *V. ruber* AF462458, *V. tubiashii* X74725, and *V. xuii* AJ316181 were included in the phylogenetic analysis (Figure 4). Phylogenetic trees were constructed using three different methods (NJ, ML, and MP). For NJ analysis, distance matrices were calculated using the Kimura two parameters correction and using MEGA version 5.0 (Tamura et al., 2011). ML and MP analysis was conducted using PHYLIP (Phylogeny Inference Package, version 3.573c, distributed by J. Felsenstein, Department of Genetics, UW, Seattle, WA, USA). Sequences corresponding to positions 86–1420 of the *E. coli* gene (NC_000913) were used in this analysis. Figure 4 represents a subset of the final tree obtained using the NJ method with 500 bootstrap replications. Nodes supported by ML and MP analyses are indicated by the bootstrap values in Figure 4.

DNAs of bacterial strains were prepared following the procedures of Marmur (1961), with minor modifications. The mol% G+C content of DNAs was determined by HPLC (Tamaoka and Komagata, 1984). DNA-DNA hybridization experiments were performed in microdilution wells using a fluorometric direct binding method described by Ezaki et al. (1988); Ezaki et al. (1989). DNA-DNA similarity data were shown as the average value of triplicate experiments. *V. brasiliensis*, *V. furnissii*, *V. fluvialis*, *V. tubiashii*, *V. hepatarius*, *V. mytili*, *V. nereis*, *V. porteresiae*, and *V. xuii* were selected as the reference species of these DNA-DNA hybridization experiments, based on the results from the MLSA molecular phylogenetic assessment and the 16S rRNA gene phylogeny of *V. tritonius* sp. nov.

A total of 62 phenotypic characteristics were determined using the standard manual characterization method established in our laboratory (Sawabe et al., 1998). The carbon assimilation test was conducted using a basal seawater medium, as previously described (Sawabe et al., 1998). These phenotypic characterizations were performed at 25°C. O/129 sensitivity was determined using the sensitivity basal agar medium (Nissui Pharmaceutical, Tokyo, Japan) at 30°C.

## Results

### The subtree incongruence test between the 9-gene and 8-gene phylogenies

**Figure 1:**
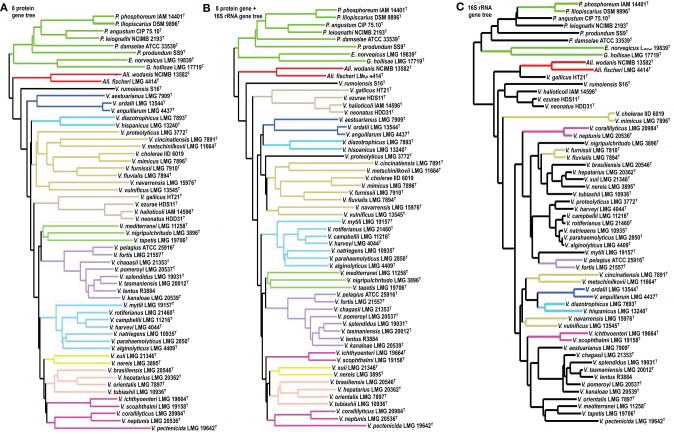
**Subtree incongruence test**. Using the 58 vibrio taxon data set reported in Sawabe et al. (2007), subtree topology was compared between 8 gene (−16S rRNA) **(A)** and 9 gene (+16S rRNA) phylogeny **(B)**. The topology was also compared between 8 gene and only 16S rRNA gene phylogeny **(C)**.

Subtrees obtained in the 8-gene phylogeny were compared to those in the 9-gene phylogeny and the 16S rRNA gene phylogeny (Figure 1). Among the 13 subtrees reconstructed in the 8-gene phylogeny, 12 were retained in the 9-gene phylogeny; the only difference observed was the inclusion of *V. proteolyticus* in the *V. cholera* subtree (Figures 1A,B). Most of the 13 subtrees in the 8-gene phylogeny corresponded to the clades that we previously proposed based on the 9-gene phylogeny (Figure 1B). The results of the subtree incongruence test using the 58 vibrio taxa data showed that the inclusion of the 16S rRNA gene sequence is not a critical factor in optimizing the vibrio phylogeny on the basis of MLSA.

### The latest vibrio phylogeny based on multilocus housekeeping protein-coding gene sequences

**Figure 2:**
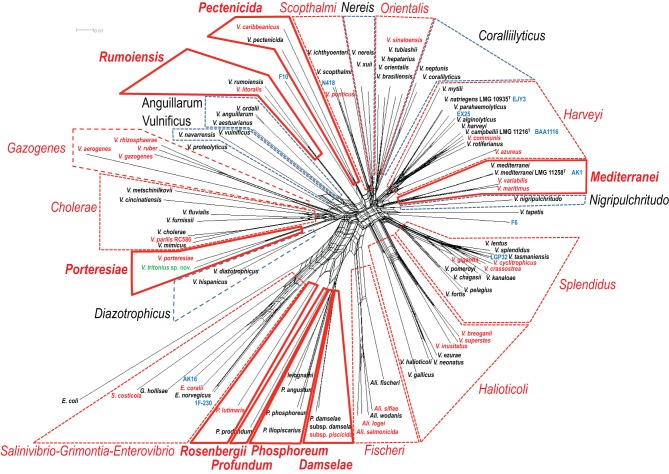
**Concatenated split network tree based on eight gene loci**. The *ftsZ*, *gapA*, *gyrB*, *mreB*, *pyrH*, *recA*, and *topA* gene sequences from 96 taxa were concatenated, and a tree was reconstructed using the SplitsTree4 program. Clades indicated by a solid red line were the “new” clades proposed in this study. Clades indicated by a dotted red line or by a dotted black line are the clades “emended” and “un-changed,” respectively.

**Figure 3:**
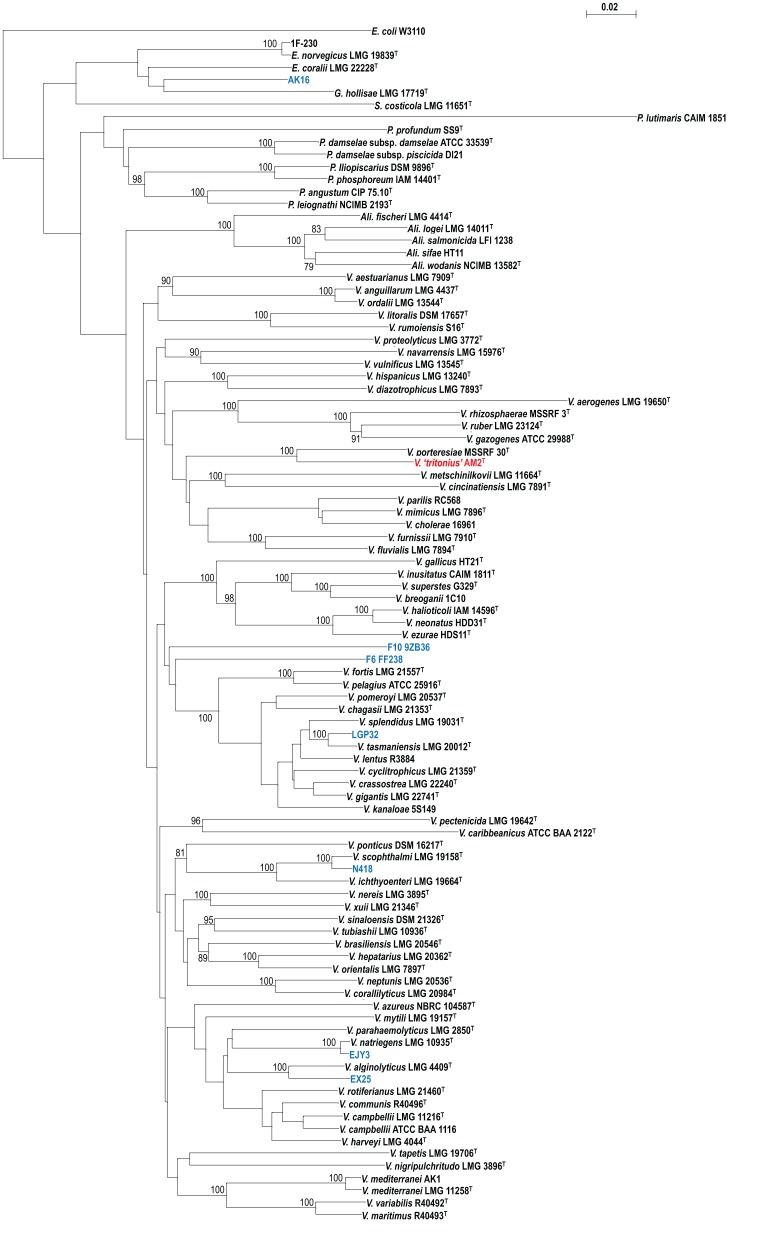
**Supertree reconstructed on the basis of the same data set drawn for Figure 2**.

WGSs of key vibrios species that were resistant to the gene amplification, e.g., *V. gazogenes*, *S. costicola*, *V. porteresiae*, *V. caribbenthicus*, are now available. This result indicated that we could use the complete set of 8 housekeeping protein-coding gene sequences currently available from 86 described vibrio species and 10 genome-sequenced *Vibrionaceae* strains for the MLSA updating of the 8-gene phylogeny, on the basis of Splits Decomposition Analysis (SDA) (Bandelt and Dress, 1992; Huson and Bryant, 2005) (Figure 2) and a supertree reconstruction (Figure 3). On the basis of SDA, we could retain the 14 distinct monophyletic clades that were previously defined, and we were able to further define 8 new clades: *Damselae*, *Mediterranei*, *Pectenicida*, *Phosphoreum*, *Profundum*, *Porteresiae*, *Rosenbergii*, and *Rumoiensis* (Figures 2, 3, Table 1). The robustness of these clades was high enough to propose their monophyly in the supertree reconstruction using three different molecular phylogenetic analyses (Figure 3). Using an 8-housekeeping protein-coding gene analysis, most of clades shared >80.5% ANI and >92% AAI, and the highest ANI (98.3%) was observed in the sequence comparison between *V. anguillarum* and *V. ordalii* (Table 1).

**Table 1:** **Newly proposed and emended clades by means of 8 gene MLSA for vibrios**.

**Clade**	**All described species included in newly proposed or newly involved species in the emended clade (in gothic)**	**Total No. of species**	**Genome sequenced strains involved in the related clade**	**8 gene MLSA concatenated identity (%)**	**8 gene-AAI (%)**	**Habitat**
**NEWLY DESCRIBED CLADE**						
***Damselae***	*P. damselae* subsp. damselae and ***P. damselae* subsp. *piscicida***	2		**96.2**	**98.7**	Seawater and fish
***Mediterranei***	***V. mediterranei, V. maritimus*, and *V. variabilis***	**3**	**AK1**	**89.5–96.3**	**98.0–99.1**	**Seawater and mucus of healthy coral**
***Pectenicida***	***V. caribbeanicus* and *V. pectenicida***	**2**		**82.8**	**96.1**	**Marine sponge and diseased bivalve larvae**
***Phosphoreum***	*P. angustum, P. iliopiscarius, P. leiognathi*, and *P. phosphoreum*	4		**87.5–95.8**	**95.7–99.3**	Seawater, luminous organ, and fish
***Profundum***	*P. profundum, P. indicum*, and *P. lipolyticum*	3		–	–	Deep sea
***Porteresiae***	***V. porteresiae* and *V. tritonius* sp. nov**.	**2**		**91.4**	**97.2**	**Wild rice and the gut of sea hare**
***Rosengergii***	***P. lutimaris*** and *P. rosenbergii*	**2**		–	–	**Seawater, coral, and tidal flat**
***Rumoiensis***	***V. litoralis* and *V. rumoiensis***	**2**		**91.5**	**98.3**	**Tidal flat sediment and drain of a fish processing factory**
**ORPHAN CLADE**						
***Tapetis***	***V. tapetis***	**1**		–	–	**Clam**
***Proteolyticus***	***V. proteolyticus***	**1**		–	–	**The intestine of isopod**
**F6**		**1**	**F6**	–	–	
**F10**		**1**	**F10**	–	–	
**EMENDED CLADE**						
*Anguillarum*	*V. anguillarum, V. aestuarianus*, and *V. ordalii*	3		**87.0–98.3**	**95.8–99.8**	Brackish, seawater, and fish
***Cholerae***	*V. cincinnatiensis, V. cholerae, V. furnissii, V. fluvialis, V. metschnikovii, V. mimicus*, and ***V. parilis***	**7**		**83.4–94.4**	**93.5–99.8**	Brackish, seawater, and clinical specimen
*Coralliilyticus*	*V. coralliilyticus* and *V. neptunis*	2		**94.2**	**99.5**	Seawater, bivalve, and rotifer
*Diazotrophicus*	*V. diazotrophicus* and *V. hispanicus*	2		**89.6**	**97.0**	Brackish and seawater
***Fischeri***	*Ali. fischeri*, ***Ali. logei, Ali. salmonicida, Ali. sifiae***, and *Ali. wodanis*	**5**		**88.7–93.5**	**94.9–99.1**	Seawater, squid, and fish
***Gazogenes***	***V. aerogenes, V. gazogenes, V. rhizosphaerae***, and ***V. ruber***	**4**		**80.5–92.2**	**92.0–99.0**	Estuary, salt marsh mud, and **rhizosphere of wild rice**
***Halioticoli***	***V. breoganii***, *V. ezurae, V. gallicus, V. halioticoli*, ***V. inusitatus***, *V. neonatus*, and ***V. superstes***	**7**		**85.7–97.5**	**95.3–99.6**	Gut of abalone and **bivalve larvae**
***Harveyi***	*V. alginolyticus*, ***V. azureus***, *V. campbellii*, ***V. communis***, *V. harveyi, V. mytili, V. natriegens, V. parahaemolyticus*, and *V. rotiferianus*	**9**	**ATCC_BAA1116, EJY3, and Ex25**	**86.7–96.0**	**96.9–99.9**	Seawater, salt marsh mud, marine animal, and **mucus of the coral**
*Nereis*	*V. nereis* and *V. xuii*	2		**89.9**	**96.9**	Seawater and srimp
*Nigripulchritudo*	*V. nigripulchritudo* and *V. penaeicida*	2		–	–	Seawater and srimp
***Orientalis***	*V. brasiliensis, V. hepatarius, V. orientalis*, ***V. sinaloensis***, and *V. tubiashii*	**5**		**88.8–93.7**	**97.1–98.9**	Brackish and seawater
***Scopthalmi***	*V. ichthyoenteri*, ***V. ponticus***, and *V. scophthalmi*	**3**	**N418**	**87.8–94.4**	**96.7–99.4**	Gut of flat fish, and **marine animal**
***Splendidus***	*V. chagasii*, ***V. crassostrea, V. cyclitrophicus***, *V. fortis*, ***V. gigantis***, *V. kanaloaei, V. lentus, V. pelagius, V. pomeroyi, V. splendidus*, and *V. tasmaniensis*	12	**LGP32**	**89.1–97.3**	**96.9–99.9**	Seawater and marine animal
*Vulnificus*	*V. navarrensis* and *V. vulnificus*	2		**86.9**	**96.5**	Sewage, seawater, eel, and oyster
***Salinivibrio-Grimontia-Enterovibrio* (Super clade)**	***S. costicola* subsp. *costicola***, *G. hollisae*, ***E. coralii***, and *E. norvegicus*	**4**	**AK16 and 1F-23**	**0 81.7–89.4**	**92.4–98.0**	Brine, human feceace, gut of turbot, and seawater

^*^Bold indicated new and/or emended information.

### New clades

*Mediterranei* consisted of three species: *V. mediterranei*, *V. maritimus*, and *V. variabilis*. The 8-gene ANI and AAI were 89.5–96.3% and 98.0–99.1%, respectively. The mol% G+C range of the clade members was 42–46.3 mol%. The genome-sequenced strain *V. mediterranei* AK1 showed 98.7% ANI and 99.9% AAI against the *V. mediterranei* type strain. Their known habitats are warm seawater and coral mucus.

*Porteresiae* consisted of *V. porteresiae* and the newly described *V. tritonius* sp. nov. Detailed information for this new species is described in the section “The bacterial taxonomical remarks of *V. tritonius* sp. nov.” below. These two species shared 91.4% ANI and 97.2% AAI. Two of the unique phenotypes in these species were an efficient H_2_ production and nitrogen fixation. While the genome sequences of these two species are highly conserved (unpublished data), they have distinct habitats (Table 1). The mol% G+C ranged from 44.2 to 45.5, and the DDH value of *V. tritonius* type strain against *V. porteresiae* type strain was 59% (Table 2).

**Table 2:** **DNA relatedness among *Vibrio tritonius* and the related vibiro species**.

**Strain**		**G+C content (moles %)**	**% Reassociation with biotinylated DNA from:**			
			***V. tritonius* JCM 16456^T^**	***V. tritonius* JCM 16457**	***V. furnissii* LMG 7910^T^**	***V. hepatarius* LMG 20362^T^**
*V. tritonius*	JCM 16456^T^	44.2	100	92	5	5
*V. tritonius*	JCM 16457	44.3	74	101	NT	23
*V. tritonius*	JCM 16458	45.5	76	104	12	11
*V. tritonius*	JCM 16459	45.2	70	100	13	14
*V. porteresiae*	MSSRF 30^T^	44.4	59^*^	NT	NT	NT
*V. furnissii*	LMG 7910^T^	52.0	12	15	100	14
*V. fluvialis*	LMG 7894^T^	49.3–50.6	20	NT	NT	NT
*V. hepatarius*	LMG 20362^T^	46.0	10	9	3	100
*V. nereis*	LMG 3895^T^	48.0	6	13	4	20
*V. tubiashii*	LMG 10936^T^	46.1	8	7	7	24

* *The reciprocal DDH value of V. tritonius JCM 16456^T^ against V. porteresiae MSSRF 30^T^ probe was 46%*.

*Pectenicida* consisted of two species, *V. caribbeanicus* and *V. pectenicida* showing 82.8% ANI and 96.1% AAI. The reported habitats were tidal flats and diseased larvae, respectively (Table 1).

*Rumoiensis* consisted of two species, *V. litoralis* and *V. rumoiensis* showing 91.5% ANI and 98.3% AAI. These species were isolated from a tidal flat and sewage from a fishery product factory, respectively (Table 1). The reported DDH value between *V. litoralis* and *V. rumoiensis* was below 7.4%.

*Damselae*, *Phosphoreum*, *Profundum*, and *Rosenbergii* were the newly proposed clades that are included in *Photobacterium* spp. These four new clades are based on ANI (87.5–96.2% in range), AAI (95.7–99.3% in range), and branch separation according to the supertree analysis in comparison with those ranges and branch separations of other *Vibrio* clades. The *Damselae* clade consisted of two subspecies of *P. damselae*. The *Rosenbergii* clade consisted of *P. lutimaris* and *P. rosenbergii*.

### Defining orphan clades

These are the clades that are formed by only one species. *V. tapetis* and *V. proteolyticus* were not grouped with any other species (Figure 2 and Table 1). The recently proposed genomospecies F6 and F10 also did not belong to any of the clades proposed in this analysis. Previously reported singletons, *V. agarivorans*, and *V. pacinii* were not included in this analysis due to the lack of some gene sequences.

### Emended clades

We can find emendations in most of the clades previously defined (Figures 2, 3, and Table 1): (1) *Cholerae* (inclusion of *V. parilis);* (2) *Fischeri* (incl. *Ali. sifae*); (3) *Gazogenes* (incl. *V. rhizosphaerae);* (4) *Halioticoli* (incl. *V. breoganii*, and *V. inusitatus*); (5) *Harveyi* (incl. *V. azureus*, and *V. communis*); (6) *Orientalis* (incl. *V. sinaloensis*); (7) *Scophthalmi* (incl. *V. ponticus*); and (8) *Splendidus* (incl. *V. cyclitrophicus, V. gigantis*, and *V. crassostrea*).

We first included the complete set of the 8-housekeeping protein-coding gene sequences of *Salinivibrio costicola* subsp. *costicola* in the MLSA for vibrio phylogeny, because its WGS data (ASAI01000001) are available. However, for the current analysis of the *Salinivibrio*/*Grimontia*/*Enterovibrio* grouping, we could use only the data set including a single species of *Salinivibrio*, 2 species of *Enterovibrio*, and a single species of *Grimontia* for the 8-gene phylogeny. As both SDA and supertree analysis showed less robustness in these genera/species grouping, we decided to tentatively define them as the *Salinivibrio-Grimontia*-*Enterovibrio* (SGE) super-clade.

### Defining the clade of genome sequenced strains

Vibrios are one of the most advanced groups in WGS analysis; currently more than 900 genomes are available in the public database (http://www.vibriobiology.net/). The MLSA of the 10 genome sequenced strains revealed: (1) LGP32 and EX25 formed a robust cluster with *V. tasmaniensis* and *V. alginolyticus*, respectively; (2) N418 and EJY3 (Roh et al., 2012) were related to *V. scopthalmi* and *V. natriegens*, respectively; and (3) The orphan positions of genomospecies F6 and F10, and AK16 (Figures 2, 3, and Table 1).

### The bacterial taxonomical remarks of *Vibrio tritonius* sp. nov.

On the basis of the 8-gene MLSA, the sea hare (*Aplysia kurodai*) isolates were highly likely to represent a new species within the family *Vibrionaceae*, more precisely within a new clade “*Porteresiae*.” Four strains of *V. tritonius* formed a robust cluster within the *Porteresiae* clade on the basis of 4-gene sequence SDA (data not shown). To confirm the taxonomic status of the sea hare strains, a standard polyphasic taxonomy was conducted.

**Figure 4:**
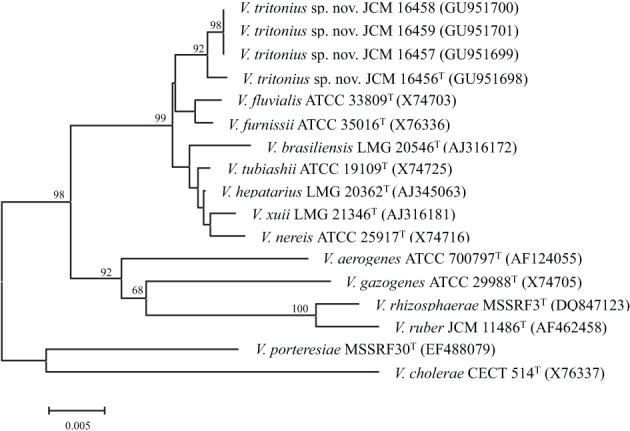
**Unrooted phylogenetic tree on the basis of 16S rRNA gene sequences**. Scale bar: 0.005 accumulated change per nucleotide. This figure combines the results of three analyses i.e., neighbor-joining, maximum parsimony, and maximum likelihood. The topology shown was obtained using neighbor-joining and 500 bootstrap replications. Percentages indicate the branches that were also obtained both in the maximum likelihood analysis (*P* < 0.01) and in the most parsimonious tree.

The results of our phylogenic analyses based on the 16S rRNA gene sequence clearly showed that these strains belong to class *Gammaproteobacteria*, and more precisely to the family *Vibrionaceae*. The closest phylogenic neighbor of the four sea hare isolates was the *V. furnissii*-*V. fluvialis* cluster (Figure 4). *V. porteresiae* was not closely related, as shown by the 16S rRNA gene sequence phylogeny. Intra-species sequence similarities of the 16S rRNA gene among *V. tritonius* sp. nov. were above 99.5%. Four strains of *V. tritonius* sp. nov. showed 98.0–98.1% similarity, and 98.1% similarity toward *V. furnissii* (X76336) and *V. fluvialis* (X74703), respectively. Sequence similarities of *V. tritonius* sp. nov. to the other phylogenetic neighbors and to gas-producing vibrios were below 98%. The 16S rRNA gene sequence similarity between *V. tritonius* sp. nov. and “*Allomonas enterica*” AJ550855 was 98.3%. The 16S rRNA gene sequences of *V. fluvialis* X74703 and “*A. enterica*“ AJ550855 were identical.

Mutual DDH experiments showed that the four strains of *V. tritonius* sp. nov., JCM 16456^T^, JCM 16457, JCM 16458, and JCM 16459, were conspecific and clearly separated from their phylogenetic neighbors, e.g., *V. porteresiae*, *V. fluvialis V. furnissii, V. tubiashii, V. hepatarius*, and *V. nereis* (Table 2). The mol% G+C content was 44.8 ± 0.6, which was within the range of the genus *Vibrio*.

### Description of *Vibrio tritonius* sp. nov

Etymology of the newly describing *Vibrio* species was provided here: *Vibrio tritonius* (tri.to'ni.us. L. masc. adj. tritonius, named after Triton (a sea-god, son of Neptune and the nymph Salacia, referring to the habitat of the bacteria).

Major phenotypic features of *V. tritonius* sp. nov. are shown in Table 3. The four sea hare strains have the major phenotypic features of the genus *Vibrio* (except for no growth on TCBS and gas production). These strains required salt for their growth, and they were motile, fermentative and oxidase positive. Apparent catalase activity was not observed. The four strains of *V. tritonius* sp. nov. were phenotypically most similar to *V. porteresiae*, but they differed from *V. porteresiae* in four traits (catalase production, and the assimilation of D-mannose, γ-aminobutyrate and pyruvate), out of 62 tested traits (Table 3). The four *V. tritonius* strains were sensitive to the vibrio-static agent O/129 (150 μ g). Positive assimilation of glucose, mannitol, gluconate, glucuronate, and xylose indicated the presence of three major carbohydrate metabolic pathways, the Embden-Meyerhof, Entner-Doudoroff, and pentose-phosphate pathways, in *V. tritonius* sp. nov. Presence of the gene set for those three central metabolic pathways of carbohydrates was supported by our preliminary WGS analysis of *V. tritonius* JCM 16456^T^ (data not shown). Phenotypic traits differentiating *V. tritonius* sp. nov. from *V. aerogenes*, which shows a gas production phenotype, included nitrate reduction, amylase production, and arginine dihydrolase activity. Inability to grow on TCBS was a common trait of *V. tritonius* sp. nov. and *V. porteresiae* (Table 3).

**Table 3:** **Phenotypic characteristics for distinguishing *Vibrio tritonius* from the related *Vibrio* species**.

**Characteristics**	***V. tritonius (n = 4)***	***V. porteresiae* MSSRF 30^T^**	***V. furnissii* LMG 7910^T^**	***V. fluvialis* LMG 7894^T^**
Gas production from D-glucose	+	+	+	−
**GROWTH AT**				
37°C	+	+	+	+
40°C	+	+	+	+
Catalase	−	+	+	+
**PRODUCTION OF**				
Amylase	−	−	+	+
Lipase	−	+w	+	+
Acetoin production	+	+	−	−
Arginine dihydrolase	−	−	+	+
Indole production	−	−	+	+
Growth on TCBS	−	−	+(Y)	+(Y)
**UTILIZATION OF**				
D-Mannose	d + (1)	−	+	+
Melibiose	d + (3)	−	−	−
Lactose	+	+	−	−
D-Glucuronate	+	+	−	+
Trehalose	−	−	+	+
Putrescine	−	−	+	−
γ-Aminobutyrate	d + (3)	−	+	+
Propionate	−	−	+	+
Maltose	+	+	+w	+
D-Glucosamine	+	+	+	+
D-Xylose	+	+	−	−
Cellobiose	+	+	+w	+

*Data were obtained under same culture condition in our laboratory. +, Positive; −, negative; w, weak reaction; d+, variable but type strain positive; d−, variable but type strain negative (numbers of positive strains are shown in parentheses). All species are motile, require Na for growth, fermentative of D-glucose, growth at 15–25 °C, oxidase-positive, produce gelatinase, DNase, and caseinase, reduce nitrate, produce acid from D-glucose, assimilate N-acetyl-D-glucosamine, D-fructose, D-galactose, gluconate, D-glucose, D-marmitol, L-glutamate, L-proline, acetate, citrate, fumarate, DL-malate, pyruvate, succinate. All species are negative for pigmentation, swarming, luminescence, growth at 4 and 45 °C, alginase production, agarase production, lysine decarboxylase, ornithine decarboxylase, requirement for organic growth factor, assimilation of D-sorbitol, aconitate, α-ketoglutarate, L-tyrosine, meso-erythritol*.

**Table 4:** **FAME dominance (%) of *Vibrio tritonius* and the related species**.

**Major fatty acids**	***V. tritonius***				*V. porteresiae* MSSRF 30^T^*^^1	***V. fluvialis* LMG 7894^T^**	***V. furnissii* LMG 7910^T^**
	**AM2^T^**	**MA12**	**MA17**	**MA35**			
C_12:0_	3.5	3.0	3.0	3.0	6.8	2.2	2.1
C_14:0_	7.7	6.5	6.1	5.7	8.2	3.5	3.6
C_16:0_	24.6	30.0	32.8	29.1	18.8	21.6	19.2
C_18:0_	1.7	0.8	1.0	0.9	0.6	2.4	3.1
C_14 : 1_ ω 7c	2.0	1.0	0.8	1.1	–	tr	0.5
C_16 : 1_ω 7c	28.5	32.0	33.7	33.0	27.5^*^^1^	36.8	32.8
C_18 : 1_ω 7c	8.3	17.8	4.8	19.1	19.2	26.9	32.2
11-methyl C_18 : 1_ω 7c	7.8	0.5	2.8	Tr	0.82	tr	tr
C_12 : 0_ 3-OH	5.3	4.6	4.0	4.6	6.4	3.2	3.2
C_14 : 0_ 3-OH	2.0	1.7	1.4	1.5	6.8^*^^1^	1.1	1.1
Unknown ECL 20.454	5.0	ND	5.6	ND	–	0.8	ND

* *1 Data from Rameshkumar et al. (2008). Values in C_16:1_ω 7c and C_14:0_ 3-OH are from the summed percentage of feature 2 and 3, respectively*.

The other phenotypic traits were also described below. No swarming cells were observed. Gas production from glucose and mannitol occurred. Cells are curved rods, with rounded ends, are 0.7–0.9 μm in diameter and 2.6–2.7 μm in length when the organism is grown on ZoBell 2216E medium; the cells occur singly on the agar. No endospores or capsules are formed. Colonies on ZoBell 2216E agar medium are beige, circular, and smooth and convex with an entire edge. Sodium ions are essential for growth. The bacterium can grow in presence of 0.5% to 6% NaCl. The bacterium is a mesophilic chemoorganotroph which grows at temperatures between 15 and 40°C. Optimal growth is observed from 25 to 30°C. Growth occurs from pH 4.5 to pH 9, and optimal growth is at pH 7.5–8.0. No growth occurs at 45°C. The bacterium is positive for acid production from glucose and mannitol; for nitrate reduction, acetoin production, and hydrolysis of gelatin, DNA and casein. The bacterium also can assimilate N-acetyl-D-glucosamine, cellobiose, D-fructose, maltose, D-mannitol, D-galactose, lactose, L-glutamate, L-proline, acetate, citrate, fumarate, DL-malate, pyruvate, and succinate. The bacterium is negative for catalase; indole production; arginine dihydrolase, lysine decarboxylase, ornithine decarboxylase, luminescence, and pigmentation; the requirement of organic growth factors; hydrolysis of agar, alginate, starch, and Tween 80; and assimilation of D-glucosamine, D-sorbitol, aconitate, α-ketoglutarate, L-tyrosine, meso-erythritol, trehalose, putrescine, propionate, and D-glucosamine. The G+C content of DNA is 44.2–45.5 mol%. The type strain is JCM 16459^T^ = LMG 25401^T^ = AM2^T^.

## Discussion

Considerable biodiversity can be found within the family *Vibrionaceae* (Gomez-Gil et al., 2014), even after the first proposal of vibrio phylogeny and evolution was inferred on the basis of MLSA in 2007 (Sawabe et al., 2007). More than 60 species of *Vibrionaceae*, with a surprising level of biodiversity, have been described since 2007. These include a marine invertebrate isolates such as coral associated vibrios (Chimetto et al., 2011; Gomez-Gil et al., 2014), introduction of nitrogen-fixing vibrios within an endophyte-like ecological niche (Rameshkumar et al., 2008), and an isolation of new vibrio species from the surface of cheese (Bleicher et al., 2010) have been reported. In addition to the increasing number of newly described vibrio species, many strains showing interesting ecophysiological features have been genome sequenced. However, taxonomic information appears to be insufficient to push the elucidation of vibrio biodiversity and evolution forward. Such a rapid progress in the study of vibrio biodiversity, genomics and evolution prompted us to update the vibrio phylogeny on the basis of MLSA. We have retrieved the complete sets of 8 house-keeping protein-coding gene sequences for 30 additional *Vibrionaceae* species including a newly described vibrio species, *V. tritonius* sp. nov., as well as for 10 as yet unnamed *Vibrio*/*Enterovibrio* spp. The MLSA led us to propose eight new clades (*Damselae*, *Mediterranei*, *Pectenicida*, *Phosphoreum*, *Profundum*, *Porteresiae*, *Rosenbergii*, and *Rumoiensis*) in the family *Vibrionaceae*, in addition to those previously proposed in the report of Sawabe et al. (2007). In 2007, *V. mediterranei*, *V. petenicida*, *V. rumoiensis*, and *P. rosenbergii* were affiliated as singlet species, but they have now been grouped. Four orphan clades (*Tapetis*, *Proteolyticus*, F6 and F10) were newly defined. More efforts are required to isolate the closest neighbors of these orphan species. Strains EJY3, EX25, N418, and LGP32 clustered robustly with *V. natriegens*, *V. alginolyticus*, *V. scopthalmi*, and *V. tasmaniensis*, but a further systematic survey is required to analyze the phylogenetic position of AK16 and 1F-230 (Figure 3).

We are still facing a lack of *Photobacterium* spp. sequences to infer their precise evolutionary history. Among the 23 described *Photobacterium* spp., we could include only half of them in this study. This situation has arisen mainly due to the “primer problems” in MLSA. Unfortunately, there are also limited numbers of WGS of *Photobacterium* spp. available in public databases. However, in this analysis, considering the results of SDA, supertree analysis, and the ANI and AAI similarity ranges in comparion to the other *Vibrio* spp. clades, we proposed four new clades for the *Photobacterium* spp.; (1) *Damselae*, (2) *Phosphoreum*, (3) *Profundum*, and (4) *Rosenbergii*. A *Salinivibrio*/*Grimontia*/*Enterovibrio* super-clade is also proposed.

In molecular phylogenetics, the use of minimum gene set is crucial to reduce time and cost, as well as to improve the accuracy, of analyses. This is of particular importance when identifying species and elucidating population structure and evolution in a super bacterial taxon such as the family *Vibrionaceae*, which has more than 140 species. The previous MLSA of 58 vibrio taxa (Sawabe et al., 2007) showed that 16S rRNA gene sequences have an extremely low species/strain discriminating power compared to the other genes tested. Therefore, before conducting the current vibrio MLSA, we evaluated whether the 16S rRNA gene data set could be eliminated from the MLSA. For this analysis, we developed a subtree incongruence test algorithm. The algorithm is a fast and reliable method for selecting subtrees that share the same topology or those that have different topologies but share the same species. The results of this analysis indicate that inclusion of 16S rRNA gene sequences is not necessary for reconstructing the vibrio phylogeny on the basis of MLSA.

We have experienced the first case in which the molecular phylogenies resulting from 16S rRNA gene sequences and from housekeeping gene sequences were largely incongruent in the species descriptions of *V. porteresiae* and *V. tritonius* (Figures 2, 4). For the affiliation of the clade of *V. porteresiae*, we used only four genes (the *pyrH*, *recA*, *rpoA*, and 16S rRNA genes), and we confirmed that *V. porteresiae* was affiliated with the *Cholerae* clade (Rameshkumar et al., 2008). The 16S rRNA gene sequence phylogeny revealed that *V. tritonius* sp. nov. was the most closely related to *V. furnissi* and *V. fulvialis* with ca. 98% sequence similarity, and *V. tritonius* sp. nov. and *V. porteresiae* were distantly related in their phylogenetic relationship (Figure 4). Less phylogenetic relatedness in the 16S rRNA gene sequence tree between both the species and the lack of housekeeping gene sequences of *V. porteresiae* prevented no direct comparison of *V. porteresiae* and *V. tritonius* sp. nov. Fortunately, the whole genome nucleotide sequences of *V. porteresieae* and *V. tritonius* sp. nov. were determined in this study, and the first direct comparison of both species by MLSA and whole genome comparison was achieved. Surprisingly, the MLSA with 8-housekeeping genes phylogeny led to the conclusion that both *Vibrio* species, *V. porteresiae* and *V. tritonius* sp. nov., share a common ancestry and that they can be proposed as a new vibrio clade, “*Porteresiae*.” Our preliminary genome comparison of both species also supported monophyly because a strong synteny was observed between both genomes (unpublished data). Incongruences between the 16S rRNA gene sequence tree and the MLSA tree were also observed in *Mediterranei* and *Pectenicida* clades (Lambert et al., 1998; Chimetto et al., 2011; Hoffmann et al., 2012) in this study. Therefore, we conclude that at present the “8-housekeeping-gene phylogeny” is the most powerful method for delineating vibrio species description/biodiversity/population study/evolution, until alternative genome-based approaches are proposed. This analysis reduces the misidentification of ancestry clades of vibrios.

The polyphasic taxonomic approach reveals that the *Aplysia* gut isolates, *V. tritonius* sp. nov., to be a novel *Vibrio* species showing gas producing ability (Figures 2–4, Tables 2, 3) and forming a robust clade, *Porteresiae*, with *V. porteresiae*. Production of gas during the fermentation of carbohydrates is not a prevalent property in the *Vibrio* genus (Shieh et al., 2000, 2003; Farmer III et al., 2005; Kumar and Nair, 2007; Rameshkumar et al., 2008). *V. aerogenes*, *V. furnissii*, *V. gazogenes*, *V. porteresiae*, *V. ruber*, and *V. rhizosphaerae* are the species in which the gas production phenotype is retained in a stable way among 98 *Vibrio* species. For this reason, gas production is an atypical property in the genus *Vibrio*. The current phylogenetic network analysis using the 8-gene MLSA confirmed that *V. aerogenes*, *V. gazogenes*, *V. rhizosphaerae*, and *V. ruber* form a robust clade, *Gazogenes* (Figure 2) (Kumar and Nair, 2007; Sawabe et al., 2007). *V. furnissii* belongs to the *Cholerae* clade. The newly described *V. tritonius* sp. nov. belongs to a new clade, *Porteresiae*. The gas compositions of *Porteresiae* clade species but also of the *Gazogenes* and *Cholerae* clades, were identical for H_2_ and CO_2_, but the H_2_ production efficiencies differ between these clades. The H_2_ production efficiency of *V. tritonius* sp. nov. and *V. porteresiae* was high and comparable to that of enterobacterial species, such as *Escherichia coli*, *Salmonella*, *Enterobacter*, and *Klebsiella* (Nakashimada et al., 2002). Our preliminary genome comparisons suggests that *V. tritonius* sp. nov. and *V. porteresiae* contain very similar gene clusters responsible for H_2_ production and nitrogen fixation machinery (unpublished data). It would be intriguing to understand how those vibrios acquire and/or lose gas producing abilities from the standpoint of both evolutionary dynamics and metabolic diversity.

In conclusion, 8-gene MLSA is a reliable tool for delineating a species and a monophyletic group or “clade.” Using the current data set reported in this study, <98% of 8-gene-concatenated nucleotide sequence identity may allow us to define a species boundary. We also showed that WGSs overcome the limitations of gene-by-gene multilocus sequencing tasks found in the MLSA for *Vibrionaceae* (primer problem, Chromosome 2 gene inclusion). In fact, we could not include *V. aerogenes*, *V. gazogenes*, *V. rhizosphaerae*, and *V. superstes* for the previous 9-gene MLSA by the primer problems (Sawabe et al., 2007), but the successful WGS for these species and the inclusion of 8 housekeeping protein gene sequences retrieved from the genome sequences can provide the better picture of current vibrio molecular phylogeny. More efforts to sequence the individual housekeeping genes and WGS of all remaining species in the family *Vibrionaceae* not involved in this study allow the possibility of elucidating the ultimate clade structure of these Vibrios. The ultimate phylogenetic trees allow us to provide the ideal phylogenetic backbone to elucidate the evolutional history, genome dynamics, and plasticity in the family *Vibrionaceae*.

### Conflict of interest statement

The authors declare that the research was conducted in the absence of any commercial or financial relationships that could be construed as a potential conflict of interest.

## Supplementary material

The GenBank accession numbers for the *ftsZ*, *gapA*, *gyrB*, *mreB*, *recA*, *rpoA*, *pyrH*, and *topA* gene sequences used in this analysis, as well as those used in Sawabe et al. (2007), are listed in Table S1.

Table S1 **List of strains and sequences accession number used for the MLSA**.

## Abbreviations

AFLP: amplified fragment length polymorphism
ANI: average nucleotide sequence identity
AAI: average amino acid identity
DDH: DNA-DNA hybridization
MLSA: multilocus sequence analysis
MLST: multilocus sequence typing
MP: maximum parsimony
NJ: neighbor joining
ML: maximum likelihood
PFGE: pulse field gel electrophoresis
RAPD: random amplified polymorphic DNA.

## References

[B1] Aanensen D. M. Spratt B. G. (2005). The multilocus sequence typing network: mlst.net. Nucleic Acids Res. 33, W728–W733 10.1093/nar/gki415 15980573 PMC1160176

[B2] Auch A. F. von Jan M. Klenk H.-P. Göker M. (2010). Digital DNA-DNA hybridization for microbial species delineation by means of genome-to-genome sequence comparison. Stand. Genomic Sci. 2, 117–134 10.4056/sigs.531120 21304684 PMC3035253

[B3] Aujoulat F. Jumas-Bilak E. Masnou A. Sallé F. Faure D. Segonds C. (2011). Multilocus sequence-based analysis delineates a clonal population of *Agrobacterium* (*Rhizobium*) *radiobacter* (*Agrobacterium tumefaciens*) of human origin. J. Bacteriol. 193, 2608–2618 10.1128/JB.00107-11 21398532 PMC3133165

[B4] Bandelt H.-J. Dress A. W. M. (1992). Split decomposition: a new and useful approach to phylogenetic analysis of distance data. Mol. Phylogenet. Evol. 1, 242–252 10.1016/1055-7903(92)90021-8 1342941

[B5] Bennett J. S. Jolley K. A. Earle S. G. Corton C. Bentley S. D. Parkhill J. (2012). A genomic approach to bacterial taxonomy: an examination and proposed reclassification of species within the genus *Neisseria*. Microbiology 158, 1570–1580 10.1099/mic.0.056077-0 22422752 PMC3541776

[B6] Bishop C. J. Aanensen D. M. Jordan G. E. Kilian M. Hanage W. P. Spratt B. G. (2009). Assigning strains to bacterial species via the internet. BMC Biol. 7:3 10.1186/1741-7007-7-3 19171050 PMC2636762

[B7] Bleicher A. Neuhaus K. Scherer S. (2010). *Vibrio casei* sp. nov., isolated from the surfaces of two French red smear soft cheeses. Int. J. Syst. Evol. Microbiol. 60, 1745–1749 10.1099/ijs.0.016493-0 19749036

[B8] Chan J. Z.-M. Halachev M. R. Loman N. J. Constantinidou C. Pallen M. J. (2012). Defining bacterial species in the genomic era: insights from the genus *Acinetobacter*. BMC Microbiol. 12:302 10.1186/1471-2180-12-302 23259572 PMC3556118

[B9] Chimetto L. A. Cleenwerck I. Moreira A. P. B. Brocchi M. Willems A. De Vos P. (2011). *Vibrio variabilis* sp. nov. and *Vibrio maritimus* sp. nov., isolated from *Palythoa caribaeorum*. Int. J. Syst. Evol. Microbiol. 61, 3009–3015 10.1099/ijs.0.026997-0 21296931

[B10] Chevenet F. Brun C. Bañuls A.-L. Jacq B. Christen R. (2006). TreeDyn: towards dynamic graphics and annotations for analyses of trees. BMC Bioinfomatics 7:439 10.1186/1471-2105-7-439 17032440 PMC1615880

[B11] Ezaki T. Hashimoto Y. Takeuchi N. Yamamoto H. Liu S.-L. Miura H. (1988). Simple genetic method to identify viridans group streptococci by colorimetric dot hybridization and fluorometric hybridization in microdilution wells. J. Clin. Microbiol. 26, 1708–1713 3183018 10.1128/jcm.26.9.1708-1713.1988 PMC266701

[B12] Ezaki T. Hashimoto Y. Yabuuchi E. (1989). Fluorometric deoxyribonucleic acid-deoxyribonucleic acid hybridization in microdilution wells as an alternative to membrane filter hybridization in which radioisotopes are used to determine genetic relatedness among bacterial strains. Int. J. Syst. Bacteriol. 39, 224–229 10.1099/00207713-39-3-224

[B13] Farmer J. J. III Janda J. M. Brenner F. W. Cameron D. N. Birkhead K. M. (2005). Genus I. *Vibrio* Pacini 1854, 411AL, in Bergey's Manual of Systematic Bacteriology, 2nd Edn., Vol. 2, eds Brenner D. J. Krieg N. R. Staley J. T. (New York, NY: Springer), 494–546

[B14] Gevers D. Cohan F. M. Lawrence J. G. Spratt B. G. Coenye T. Feil E. J. (2005). Re-evaluating prokaryotic species. Nat. Microbiol. 3, 733–739 10.1038/nrmicro1236 16138101

[B15] Gomez-Gil B. Thompson C. C. Matsumura Y. Sawabe T. Iida T. Christen R. (2014). Family Vibrionaceae (Chapter 225), in The Prokaryotes, 4th Edn., eds Rosenberg E. DeLong E. F. Thonpson F. L. Lory S. Stackebrandt E. (New York, NY: Springer), 88

[B16] Goris J. Konstantinidis K. T. Klappenbach J. A. Coenye T. Vandamme P. Tiedje J. M. (2007). DNA-DNA hybridization values and their relationship to whole-genome sequence similarities. Int. J. Syst. Evol. Microbiol. 57, 81–91 10.1099/ijs.0.64483-0 17220447

[B17] Grimes D. J. Johnson C. N. Dillon K. S. Flowers A. R. Noriea N. F. III Berutti T. (2009). What genomic sequence information has revealed about vibrio ecology in the ocean-a review. Microb. Ecol. 58, 447–460 10.1007/s00248-009-9578-9 19727929

[B18] Guénoche A. (2013). Multiple consensus trees: a method to separate divergent genes. BMC Bioinformatics 14:46 10.1186/1471-2105-14-46 23394478 PMC3599424

[B19] Haley B. J. Grim C. J. Hasan N. A. Choi S.-Y. Chun J. Brettin T. S. (2010). Comparative genomic analysis reveals evidence of two novel *Vibrio* species closely related to *V*. cholera. BMC Microbiol. 10:154 10.1186/1471-2180-10-154 20507608 PMC2889950

[B20] Hoffmann M. Monday S. R. Allard M. W. Strain E. A. Whittaker P. Naum M. (2012). *Vibrio caribbeanicus* sp. nov., isolated from the marine sponge Scleritoderma cyanea. Int. J. Syst. Evol. Microbiol. 62, 1736–1743 10.1099/ijs.0.032375-0 21930677

[B21] Huerta-Cepas J. Dopazo J. Gabaldón T. (2010). ETE: a python environment for tree exploration. BMC Bioinformatics 11:24 10.1186/1471-2105-11-24 20070885 PMC2820433

[B22] Huson D. H. Bryant D. (2005). Application of phylogenetic networks in evolutionary studies. Mol. Biol. Evol. 23, 254–267 10.1093/molbev/msj030 16221896

[B23] Konstantinidis K. T. Tiedje J. M. (2005). Genomic insights that advance the species definition for prokaryotes. Proc. Natl. Acad. Sci. U.S.A. 102, 2567–2572 10.1073/pnas.0409727102 15701695 PMC549018

[B24] Kumar R. N. Nair S. (2007). *Vibrio rhizosphaerae* sp. nov., a novel red-pigmented species that antagonizes phytopathogenic bacteria. Int. J. Syst. Evol. Microbiol. 57, 2241–2246 10.1099/ijs.0.65017-0 17911290

[B25] Lambert C. Nicolas J. L. Cilia V. Corre S. (1998). *Vibrio pectenicida* sp. nov., a pathogen of scallop (Pecten maximus) larvae. Int. J. Syst. Bacteriol. 48, 481–487 10.1099/00207713-48-2-481 9731288

[B26] Larkin M. A. Blackshields G. Brown N. P. Chenna R. McGettigan P. A. McWilliam H. (2007). Clustal W and Clustal X version 2.0. Bioinformatics 23, 2947–2948 10.1093/bioinformatics/btm404 17846036

[B27] Lin B. Wang Z. Malanoski A. P. O'Grady E. A. Wimpee C. F. Vuddhakul V. (2010). Comparative genomic analyses identify the *Vibrio harveyi* genome sequenced strains BAA-1116 and HY01 as *Vibrio campbellii*. Environ. Microbiol. Rep. 2, 81–89 10.1111/j.1758-2229.2009.00100.x 20686623 PMC2912166

[B28] Maiden M. C. J. Bygraves J. A. Feil E. Morelli G. Russell J. E. Urwin R. (1998). Multilocus sequence typing: a portable approach to the identification of clones within populations of pathogenic microorganisms. Proc. Natl. Acad. Sci. U.S.A. 95, 3140–3145 10.1073/pnas.95.6.3140 9501229 PMC19708

[B29] Marmur J. (1961). A procedure for the isolation of deoxyribonucleic acid from microorganisms. J. Mol. Biol. 3, 208–218 10.1016/S0022-2836(61)80047-8

[B30] Nakashimada Y. Rachman M. A. Kakizono T. Nishio N. (2002). Hydrogen production of *Enterobacter aerogenes* altered by extracellular and intracellular redox states. Int. J. Hydrogen Energy 27, 1399–1405 10.1016/S0360-3199(02)00128-3

[B31] Oppenheimer C. H. ZoBell C. E. (1952). The growth and viability of sixty-three species of marine bacteria as influenced by hydrostatic pressure. J. Mar. Res. 11, 10–18

[B32] Preheim S. P. Timberlake S. Pols M. F. (2011). Merging taxonomy with ecological population prediction in a case study of *Vibrionaceae*. Appl. Environ. Microbiol. 77, 7195–7206 10.1128/AEM.00665-11 21873482 PMC3194886

[B33] Rameshkumar N. Fukui Y. Sawabe T. Nair S. (2008). *Vibrio porteresiae* sp. nov., a diazotrophic bacterium isolated from a mangrove-associated wild rice (Porteresia coarctata Tateoka). Int. J. Syst. Evol. Microbiol. 58, 1608–1615 10.1099/ijs.0.65604-0 18599703

[B34] Richer M. Rosselló-Móra R. (2009). Shifting the genomic gold standard for the prokaryotic species definition. Proc. Natl. Acad. Sci. U.S.A. 106, 19126–19131 10.1073/pnas.0906412106 19855009 PMC2776425

[B35] Roger F. Marchandin H. Jumas-Bilak E. Kodjo A. the colBVH study group Lamy B. (2012). Multilocus genetics to reconstruct aeromonad evolution. BMC Microbiol. 12:62 10.1186/1471-2180-12-62 22545815 PMC3487998

[B36] Roh H. Yun E. J. Lee S. Ko H. J. Kim S. Kim B. Y. (2012). Genome sequence of *Vibrio* sp. strain EJY3, an agarolytic marine bacterium metabolizing 3,6-anhydro-L-galactose as a sole carbon source. J. Bacteriol. 194, 2773–2774 10.1128/JB.00303-12 22535948 PMC3347216

[B37] Sawabe T. Kita-Tsukamoto K. Thompson F. L. (2007). Inferring the evolutionary history of vibrios by means of multilocus sequence analysis. J. Bacteriol. 189, 7932–7936 10.1128/JB.00693-07 17704223 PMC2168739

[B38] Sawabe T. Koizumi S. Fukui Y. Nakagawa S. Ivanova E. P. Kita-Tsukamoto K. (2009). Mutation is the main driving force in the diversification of the *Vibrio splendidus* clade. Microbes Environ. 24, 281–285 10.1264/jsme2.ME09128 21566386

[B39] Sawabe T. Sugimura I. Ohtsuka M. Nakano K. Tajima K. Ezura Y. (1998). *Vibrio halioticoli* sp. nov., a non-motile alginolytic marine bacterium isolated from the gut of abalone Haliotis discus hannai. Int. J. Syst. Bacteriol. 48, 573–580 10.1099/00207713-48-2-573 9731299

[B40] Shieh W. Y. Chen A.-L. Chiu H.-H. (2000). *Vibrio aerogenes* sp. nov., a facultatively anaerobic marine bacterium that ferments glucose with gas production. Int. J. Syst. Evol. Microbiol. 50, 321–329 10.1099/00207713-50-1-321 10826819

[B41] Shieh W. Y. Chen Y.-W. Chaw S.-M. Chiu H.-H. (2003). *Vibrio ruber* sp. nov., a red, facultatively anaerobic, marine bacterium isolated from sea water. Int. J. Syst. Evol. Microbiol. 53, 479–484 10.1099/ijs.0.02307-0 12710616

[B42] Stackebrandt E. Frederiksen W. Garrity G. M. Grimont P. A. D. Kämpfer P. Maiden M. C. J. (2002). Report of the *ad hoc* committee for the re-evaluation of the species definition in bacteriology. Int. J. Syst. Evol. Microbiol. 52, 1043–1047 10.1099/ijs.0.02360-0 12054223

[B43] Staley J. T. (2006). The bacterial species dilemma and the genomic–phylogenetic species concept. Philos. Trans. R. Soc. B Biol. Sci. 361, 1899–1909 10.1098/rstb.2006.1914 17062409 PMC1857736

[B44] Sugawara H. Ohyama A. Mori H. Kurokawa K. (2009). Microbial Genome Annotation Pipeline (MiGAP) for diverse users, in 20th International Conference on Genome Informatics (Kanagawa), S-001, 1–2

[B45] Tamaoka J. Komagata K. (1984). Determination of DNA base composition by reversed-phase high-performance liquid chromatography. FEMS Microbiol. Lett. 25, 125–128 10.1111/j.1574-6968.1984.tb01388.x

[B46] Tamura K. Peterson D. Peterson N. Stecher G. Nei M. Kumar S. (2011). MEGA5: molecular evolutionary genetics analysis using maximum likelihood, evolutionary distance, and maximum parsimony methods. Mol. Biol. Evol. 28, 2731–2739 10.1093/molbev/msr121 21546353 PMC3203626

[B47] Thompson C. C. Vicente A. C. Souza R. C. Vasconcelos A. T. Vesth T. Alves N. Jr. Ussery D. W. (2009). Genomic taxonomy of Vibrios. BMC Evol. Biol. 9:258 10.1186/1471-2148-9-258 19860885 PMC2777879

[B48] Thompson F. L. Gevers D. Thompson C. C. Dawyndt P. Naser S. Hoste B. (2005). Phylogeny and molecular identification of Vibrios on the basis of multilocus sequence analysis. Appl. Environ. Microbiol. 71, 5107–5115 10.1128/AEM.71.9.5107-5115.2005 16151093 PMC1214639

[B49] Thompson F. L. Gomez-Gil B. Vasconcelos A. T. R. Sawabe T. (2007). Multilocus sequence analysis reveals that *Vibrio harveyi* and *V. campbellii* form distinct species. Appl. Environ. Microbiol. 73, 4279–4285 10.1128/AEM.00020-07 17483280 PMC1932783

[B50] Thompson F. L. Hoste B. Vandemeulebroecke K. Swings J. (2001). Genomic diversity amongst *Vibrio* isolated from different sources determined by fluorescent amplified fragment length polymorphism. Syst. Appl. Microbiol. 24, 520–538 10.1078/0723-2020-00067 11876360

[B52] Wayne L. G. Brenner D. J. Colwell R. R. Grimont P. A. D. Kandler O. Krichevsky M. I. (1987). Report of the *Ad Hoc* Committee on reconciliation of approaches to bacterial systematics. Int. J. Syst. Bacteriol. 37, 463–464 10.1099/00207713-37-4-463

