# Corrigendum: Updating the Vibrio clades defined by multilocus sequence phylogeny: proposal of eight new clades, and the description of *Vibrio tritonius* sp. nov.

**DOI:** 10.3389/fmicb.2014.00583

**Published:** 2014-11-04

**Authors:** Tomoo Sawabe, Yoshitoshi Ogura, Yuta Matsumura, Gao Feng, A. K. M. Rohul Amin, Sayaka Mino, Satoshi Nakagawa, Toko Sawabe, Ramesh Kumar, Yohei Fukui, Masataka Satomi, Ryoji Matsushima, Fabiano L. Thompson, Bruno Gomez Gil, Richard Christen, Fumito Maruyama, Ken Kurokawa, Tetsuya Hayashi

**Affiliations:** ^1^Laboratory of Microbiology, Faculty of Fisheries Sciences, Hokkaido UniversityHakodate, Japan; ^2^Division of Genomics and Bioenvironmental Science, Frontier Science Research Center, University of MiyazakiMiyazaki, Japan; ^3^Department of Food and Nutrition, Hakodate Junior CollegeHakodate, Japan; ^4^National Institute for Interdisciplinary Science and Technology (CSIR)Kerala, India; ^5^National Research Institute of Fisheries Science, Fisheries Research AgencyYokohama, Japan; ^6^Department of Genetics, Center of Health Sciences, Federal University of Rio de Janeiro (UFRS)Rio de Janeiro, Brazil; ^7^A.C. Unidad Mazatlán, CIADMazatlán, México; ^8^CNRS UMR 7138, Systématique-Adaptation-EvolutionNice, France; ^9^Systématique-Adaptation-Evolution, Université de Nice-Sophia AntipolisNice, France; ^10^Graduate School of Medical and Dental Sciences, Tokyo Medical and Dental UniversityTokyo, Japan; ^11^Earth-Life Science Institute, Tokyo Institute of TechnologyTokyo, Japan

**Keywords:** vibrios, *Vibrionaceae*, multilocus sequence analysis, evolution, housekeeping protein gene, *Vibrio tritonius*

Strain numbers of *Vibrio tritonius* des-cribed on pages 3 and 10 were incorrect.

Correct strain numbers are;

JCM 16456^T^=LMG 25401^T^=AM2^T^,

JCM 16457=LMG 25404=MA35,

JCM 16458=LMG 25403=MA17, and

JCM 16459=LMG 25402=MA12.

Corrected description is follows;

**Description of *Vibrio tritonius* sp. nov**. Etymology of the newly describing *Vibrio* species was provided here: *Vibrio tritonius* (tri.to'ni.us. L. masc. adj. tritonius, named after Triton (a sea-god, son of Neptune and the nymph Salacia, referring to the habitat of the bacteria).

Major phenotypic features of *V. tritonius* sp. nov. are shown in Table 3. The four sea hare strains have the major phenotypic features of the genus *Vibrio* (except for no growth on TCBS and gas production). These strains required salt for their growth, and they were motile, fermentative, and oxidase positive. Apparent catalase activity was not observed. The four strains of *V. tritonius* sp. nov. were phenotypically most similar to *V. porteresiae*, but they differed from *V. porteresiae* in four traits (catalase production, and the assimilation of D-mannose, γ-aminobutyrate, and pyruvate), out of 62 tested traits (**Table 3**). The four *V. tritonius* strains were sensitive to the vibrio-static agent O/129 (150 μg). Positive assimilation of glucose, mannitol, gluconate, glucuronate, and xylose indicated the presence of three major carbohydrate metabolic pathways, the Embden-Meyerhof, Entner-Doudoroff, and pentose-phosphate pathways, in *V. tritonius* sp. nov. Presence of the gene set for those three central metabolic pathways of carbohydrates was supported by our preliminary WGS analysis of *V. tritonius* JCM 16456^T^ (data not shown). Phenotypic traits differentiating *V. tritonius* sp. nov. from *V. aerogenes*, which shows a gas production phenotype, included nitrate reduction, amylase production, and arginine dihydrolase activity. Inability to grow on TCBS was a common trait of *V. tritonius* sp. nov. and *V. porteresiae* (**Table 3**).

The other phenotypic traits were also described below. No swarming cells were observed. Gas production from glucose and mannitol occurred. Cells are curved rods, with rounded ends, are 0.7–0.9 μm in diameter and 2.6–2.7 μm in length when the organism is grown on ZoBell 2216E medium; the cells occur singly on the agar. No endospores or capsules are formed. Colonies on ZoBell 2216E agar medium are beige, circular, and smooth and convex with an entire edge. Sodium ions are essential for growth. The bacterium can grow in presence of 0.5%–6% NaCl. The bacterium is a mesophilic chemoorganotroph which grows at temperatures between 15 and 40°C. Optimal growth is observed from 25 to 30°C. Growth occurs from pH 4.5 to pH 9, and optimal growth is at pH 7.5–8.0. No growth occurs at 45°C. The bacterium is positive for acid production from glucose and mannitol; for nitrate reduction, acetoin production, and hydrolysis of gelatin, DNA, and casein. The bacterium also can assimilate N-acetyl-D-glucosamine, cellobiose, D-fructose, maltose, D-mannitol, D-galactose, lactose, L-glutamate, L-proline, acetate, citrate, fumarate, DL-malate, pyruvate, and succinate. The bacterium is negative for catalase; indole production; arginine dihydrolase, lysine decarboxylase, ornithine decarboxylase, luminescence, and pigmentation; the requirement of organic growth factors; hydrolysis of agar, alginate, starch, and Tween 80; and assimilation of D-glucosamine, D-sorbitol, aconitate, α-ketoglutarate, L-tyrosine, meso-erythritol, trehalose, putrescine, propionate, and D-glucosamine. The G+C content of DNA is 44.2–45.5 mol%. The type strain is JCM 16456^T^=LMG 25401^T^=AM2^T^.

## Conflict of interest statement

The authors declare that the research was conducted in the absence of any commercial or financial relationships that could be construed as a potential conflict of interest.

